# Clinical, Laboratory, and Electrocardiographic Findings in Colchicine Toxicity: 10 Years of Experience

**DOI:** 10.3389/fmed.2022.872528

**Published:** 2022-05-19

**Authors:** Mehdi Sheibani, Nasim Zamani, Amir Houshang Gerami, Hossein Akhondi, Hossein Hassanian-Moghaddam

**Affiliations:** ^1^Cardiovascular Research Center, Shahid Beheshti University of Medical Science, Tehran, Iran; ^2^Department of Clinical Toxicology, Loghman-Hakim Hospital, School of Medicine, Shahid Beheshti University of Medical Sciences, Tehran, Iran; ^3^Social Determinants of Health Research Center, Shahid Beheshti University of Medical Sciences, Tehran, Iran; ^4^School of Medicine, Shahid Beheshti University of Medical Science, Tehran, Iran; ^5^Department of Internal Medicine, University of Central Florida and Florida State University, Orlando, FL, United States

**Keywords:** colchicine toxicity, colchicine poisoning, intoxication, ECG manifestations, mortality

## Abstract

**Background:**

We aimed to investigate the clinical, laboratory, and electrocardiographic (ECG) findings of colchicine poisoning and to evaluate if there is a correlation between them and the two major outcomes of this toxicity which are respiratory/cardiovascular failure and death.

**Materials and Methods:**

Medical records of 34 colchicine-intoxicated patients that were treated in our center during the past 10 years were retrospectively evaluated. The patient's clinical presentation, vital signs, laboratory tests, ECGs, and outcomes were reviewed.

**Results:**

Abdominal pain, and hypotension at presentation had significant correlation with mortality (*p* = 0.003, OR: 2.2 [4.1, 7.9], *p* = 0.029, OR: 13.0 [1.5, 111.8]). Mortality significantly occurred in those with sinus tachycardia, hypokalemia, metabolic acidosis, and impaired liver and kidney function tests (*p*-values = 0.025, 0.007, 0.04, and 0.008, respectively). All the patients had some ECG abnormalities. Most frequent ECG abnormalities were pathologic ST segment elevation and depression (70%), left atrial enlargement (48%), and sinus tachycardia (37%), PR elevation in aVR lead (37%), and T wave inversion (37%).

**Conclusions:**

Colchicine toxicity is a dangerous entity regarding the cardiovascular events and requires close general and cardiac monitoring.

## Introduction

Colchicine is a natural alkaloid compound primarily used to treat gouty arthritis, familial Mediterranean fever, amyloidosis, and severe constipation refractory to standard medical therapy (1, 2). It has also been suggested as a potential treatment for Bechet's disease, pericarditis, and atrial fibrillation after cardiac surgery. The mechanism of action is to forestall microtubule aggregation and subsequently down-regulate microtubule-based inflammatory cycles including chemotaxis (3). Colchicine is available as intravenous injection, solution, tablet, and capsule (4).

In addition to the therapeutic effects, colchicine can also be toxic due to its narrow therapeutic range. Acute overdose of colchicine is rare but constitutes one of the most serious clinical toxicology emergencies with high morbidity and mortality rates (5). Supportive care is the cornerstone of treatment and Fab fragment antibodies that are used as experimental antidote are not commercially available, making it a potentially dangerous entity (6, 7).

The toxicity of colchicine seems to be dose-dependent (6, 8). It is generally accepted that the maximum safe dose of colchicine is about 1.2 mg/day (9). Colchicine is therapeutic at 0.015 mg/kg, toxic at 0.1 mg/kg and fatal at 0.8 mg/kg (6, 7). However, death and severe sequelae are well documented at lower doses (7, 8). Fatal overdose of colchicine has been delineated in several articles where blood or plasma levels varied from 10 to 250 ng/ml (1, 2, 10).

Abdominal pain, nausea, vomiting, and diarrhea are the principal clinical manifestations of colchicine toxicity. Multi-Organ involvement is the major cause of death. In the first 10-24 h after ingestion, patients present with symptoms mimicking gastroenteritis, which are absent for intravenous users. 24 h to 7 days after ingestion is the time for multi-organ presentation and recovery follows after 7 days (7).

Cardiogenic shock secondary to direct colchicine-induced cardiac toxicity is another major cause of death. Colchicine causes direct cellular toxicity that ends up in decreased myocardial contractility (11). It is also considered to interfere with cardiac pulse generation and conduction. However, the mechanism behind the abnormal rhythms is not well understood (12, 13).

We designed this study to evaluate the different aspects of colchicine toxicity as related to the clinical, laboratory, and ECG manifestations and to see which one correlates more with respiratory/cardiovascular failure and death. Also, we aimed to find more about ECG changes that can happen from cardiac toxicity.

## Materials and Methods

### Study Design

Retrospective single center evaluation of colchicine toxic cases who were referred to the emergency department of Loghman-Hakim Hospital—the main referral toxicology center in Tehran, Iran—between January 2011 and January 2021. The study was reviewed and approved by Institutional Review Board of Shahid Beheshti University of Medical Sciences (IR.SBMU.MSP.REC.1398.116).

### Inclusion and Exclusion Criteria

Colchicine-poisoned patients either as the sole agent of toxicity or as the main cause of toxicity were included. Colchicine poisoning was confirmed based on the patient history as well as signs and symptoms of the toxicity on presentation. Symptoms such as abdominal pain, nausea, vomiting were noted and subsequent manifestations such as bone marrow suppression and alopecia were included. Evaluation of serum colchicine level was not available in our center.

Patients with history of ischemic heart disease, heart failure, severe valvular heart disease, and severe renal failure [Glomerular Filtration Rate (GFR) < 30 ml/min] (14) or hepatic failure [cirrhosis or Aspartate Aminotransferase/alanine aminotransferase (AST/ALT) > 1,000 U/L] (15) were excluded. Patients who had concomitantly overdosed on a cardiotoxic medication or substances such as amphetamine or methadone were also excluded.

### Variables

Patients' demographic characteristics and point of care signs and symptoms, dosage of colchicine causing the toxicity, ECG, and lab tests were reviewed. Respiratory status and the need for mechanical ventilation was noted. Mortality was noted.

Point of care ECGs were evaluated by a cardiologist using the standard normal ranges for each (16) (Table 1). Heart rate, rhythm, axis, atrial abnormalities, PR interval, PR deviation, pathologic Q wave, QRS duration, ventricular enlargement, ST-T abnormalities, J-point elevation, early repolarization, Brugada pattern, abnormal U wave, and corrected QT intervals (QTc) were all evaluated.

**Table 1:** Definitions used for determination of abnormal variables.

**Terminology/abbreviation**	**Definition**
Tachycardia^a^ Bradycardia^a^	Heart rate > 100/min Heart rate <60/min
LAD (Left axis deviation) RAD (Right axis deviation)	Mean QRS axis more positive than + 90° (usually with rS in lead I) Mean QRS axis more negative than – 30° (usually with rS in lead II)
LAHB LPHB	QRS axis between – 45 and – 90° and qR pattern in AVL QRS axis between + 90 and + 180° and rS in I and AVL and qR in III and AVF
LA abnormality	P wave duration in lead II > 120 ms or Increased duration and depth of terminal negative portion of p wave in lead V1
RA abnormality	Peaked P wave with amplitudes in lead II > 0.25 mV or Increased area under initial positive part of P wave in lead V1 to >0.06 mm s
PVC PAC	Premature ventricular contraction Premature atrial contraction
PR deviation	Deviation of PR segment from isoelectric line (segment between T and P waves)
First degree AV block	All atrial impulses conducted to the ventricle with prolonged PR (> 200 ms)
PR interval	From the onset of P wave to the onset of QRS
QRS duration LBBB RBBB	Duration from the onset to the end of QRS QRS duration ≥ 120 ms and absent or small r + deep S in lead V1 QRS duration ≥ 120 ms and rsr′, rsR′orrSR′ in leads V1 and V2
Pathologic Q wave	Q-wave duration ≥ 0.03 s and > 0.1 mV deep
LVH RVH	Evaluated by Sokolow–lyon and cornel voltage criteria R in V1 ≥ 0.7 mV, QR in V1, R/S in V1 > 1 and R ≥ 0.5 mV, S in V6 > 0.7 mV
Poor r progression	R wave height ≤ 3 mm in V3
ST elevation Cup like Dom like	ST segment elevation from isoelectric line (segment between T and P waves) ST segment concave up ST segment concave
Specific ST elevation	A ST elevation with characteristics of acute myocardial injury
ST depression	ST segment depression from isoelectric line (segment between T and P waves)
ST abnormality	ST elevation + ST depression
ST shortening	Shortening of ST segment without elevation or depression
J point elevation	Elevation of J point (point of junction between QRS and ST segment)
Early repolarization	Elevation of the QRS-ST junction (J point) often associated with a late QRS slurring or notching (J wave)
Brugada pattern	Coved type ST elevation with at least 2 mm (0.2 mV) J-point elevation and a gradually descending ST segment followed by a negative T-wave
Primary T wave inversion	Primary T-wave abnormalities due to alterations in myocardial cellular electrophysiology not due to LBBB or LVH (secondary)
Other T wave abnormalities	Biphasic T, Tall T, and camel hump T
Pathologic U wave	U wave height > 1–2 mm, U wave height > 25% of T wave height, inverted U wave (different direction from adja- cent T wave)
QTc (corrected QT)	QT interval corrected with heart rate by bazett's formula: QTc = QT/√*^*RR*^*
QTc prolongation	QTc > 440 ms in men and > 460 ms in women

*Definitions and Measuring Criteria for ECG Variables (17)*.

a *Calculation for pediatrics were based on normal range for their age*.

### Statistics

Quantitative variables were given as mean (±SD) and median [interquartile range; IQR] for variables with normal and non-normal distribution, respectively. Data was analyzed using Pearson Chi-square, Fisher's exact test, *t*-test, and Mann-Whitney *U*-test. Measure of association between binomial exposure and outcome was expressed using odds ratio (OR) and 95% confidence interval. Roc curve was used to define the best cut-off for instantaneous sensitivity and specificity in continuous variable with significant association with mortality. Data was analyzed using statistical package for social sciences (SPSS) software version 27. *P*-values < 0.05 were considered to be statistically significant.

## Results

A total of 34 colchicine-intoxicated patients were evaluated. There were no patients with known cardiovascular or other significant comorbidities. Sixteen patients had intentionally overdosed to attempt suicide; two had accidentally overdosed on colchicine, and in other 16, the intention was not clear.

### History and Primary Symptoms

Almost all patients were conscious upon arrival and were able to communicate with the physician and report symptoms. Subsequently, some gradually lost consciousness.

The most common presentations were nausea in 28 cases (82%) and vomiting in 24 (70%). Twelve patients (35%) had abdominal pain and 11 (32%) had diarrhea. Eleven (35%) reported dizziness as the main presentation. Other less frequent clinical presentations included dyspnea in 4 (12%), limb numbness in 3 (9%), facial paresthesia/numbness in 3 (9%), headache in 3 (9%), tremor in 2 (6%), and ataxia in one patient. The patients' characteristics are summarized in Tables 2, 3.

**Table 2:** Patient history and primary symptoms in mortality and mechanical ventilation groups (qualitative variables).

**Variable**		**Mortality**		***P*-value**	**OR (95% CI)**	**Intubation**		***P*-value**	**OR (95% CI)**
		**Yes (*n* = 5)**	**No (*n* = 29)**			**Yes (*n* = 5)**	**No (*n* = 29)**	
Gender *n* (%)	Male	2 (40)	11 (37.9)	1	1.1 (0.2, 7.6)	3 (60)	10 (34.5)	0.348	2.8 (0.4, 19.9)
	Female	3 (60)	18 (62.1)			2 (40)	19 (65.5)		
Pure or mix *n* (%)	Pure	4 (80)	19 (65.5)	1	2.1 (0.2, 21.5)	4 (80)	19(65.5)	1	2.1 (0.2, 21.5)
	Mixed	1 (20)	10 (34.5)			1 (20)	10 (34.5)		
In hospital mortality cause *n* (%)	Asystole	3 (60)	29 (100)	0	-	2 (40)	1 (3.4)	0	-
	VT	1 (20)	0			1 (20)	0		
	Bradycardia	1 (20)	0			1 (20)	0		
PMH HTN *n* (%)	Normotensive	4 (80)	28 (96.6)	0.276	0.1 (0.01, 2.8)	4 (80)	28 (96.6)	0.276	0.1 (0.01, 2.8)
	Unknown	1 (20)	1 (3.4)			1 (20)	1 (3.4)		
PMH DM *n* (%)	Nondiabetic	4 (80)	28 (96.6)	0.276	0.1 (0.01, 2.8))	4 (80)	28 (96.6)	0.276	0.1 (0.01, 2.8)
	Unknown	1 (20)	1 (3.4)			1 (20)	1 (3.4)		
PMH FMF *n* (%)		0	3 (10.3)	1	1.1 (1.0, 1.2)	0	5 (100)	1	1.1 (1.0, 1.2)
No Cardiac FH *n* (%)		5 (100)	29 (100)			5 (100)	29 (100)	-	-
FH Rheumatologic *n* (%)		2 (40)	4 (13.8)	0.205	4.2 (0.5, 33. 3)	3 (60)	3 (10.3)	0.029	13 (1.5, 111.8)
Usage Cause *n* (%)	Suicidal	2 (40)	14 (48.3)	0.735	-	2 (40)	0	0.735	-
	Unintentionally	0	2 (6.9)			0	2(6.9)		
	Unknown	3 (60)	13 (44.8)			3 (60)	2 (5.9)		
Consciousness when admitted *n* (%)	Conscious	4 (80)	29 (100)	0.147	0.8 (0.5, 1.2)	5 (100)	28 (96.6)	1	1.0 (1.0, 1.1)
	L.O.C	1(20)	0			0	1 (3.4)		
Primary presentation dizziness *n* (%)		1 (20)	10 (34.5)	1	0.5 (0.1, 4.8)	2 (40)	9 (31)	1	1.5 (0.2, 10.5)
Primary presentation nausea *n* (%)		5 (100)	23 (79.3)	0.559	1.3 (1.0, 1.5)	5 (100)	23 (79.3)	0.559	1.3 (1.0, 1.5)
Primary presentation vomiting *n* (%)		5 (100)	19 (65.5)	0.291	1.5 (1.2, 2.0)	5 (100)	19(65.5)	0.291	1.5 (1.2, 2.0)
Primary presentation abdominal pain *n* (%)		5 (100)	7 (24.1)	0.003	4.1 (2.2, 7.9)	4 (80)	8 (27.6)	0.042	10.5 (1.0, 108.7)
Primary presentation nonPO *n* (%)		5 (100)	19 (65.5)	0.291	1.5 (1.2, 2.0)	5 (100)	19 (65.5)	0.291	1.5 (1.2, 2.0)
Primary presentation dyspnea *n* (%)		2 (40)	2 (6.9)	0.094	9.0 (0.9, 89.3)	3 (60)	1(3.4)	0.006	42 (2.9, 612.3)
Primary presentation diarrhea *n* (%)		3 (60)	8 (27.6)	0.3	3.9 (0.5, 28.1)	2(40)	9(31)	1	1.5 (0.2, 10.5)
Primary presentation depressed mood *n* (%)		0	4 (13.8)	1	1.2 (1.0, 1.3)	1 (20)	3 (10.3)	0.488	2.2 (0.2, 26.3)
Primary presentation tremor *n* (%)		0	2 (6.9)	1	1.074 (0.973, 1.186)	0	2 (6.9)	1	1.1 (1.0, 1.2)
Primary presentation limb numbness *n* (%)		2 (40)	1 (3.4)	0.05	18.667 (1.3, 272.1)	2 (40)	1 (3.4)	0.05	18.7 (1.3, 272.1)
Primary presentation facial paresthesia/numbness *n* (%)		1 (20)	2 (6.9)	0.389	3.4 (0.2, 46.4)	1 (20)	2 (6.9)	0.389	3.4 (0.2, 46.4)
Primary presentation ataxia *n* (%)		1 (20)	0	0.147	0.8 (0.5, 1.2)	1 (20)	0	0.147	0.8 (0.5, 1.2)
Primary presentation headache *n* (%)		1 (20)	2 (6.9)	0.389	3.4 (0.2, 46.4)	1 (20)	2 (6.9)	0.389	3.4 (0.2, 46.4)
Intubation *n* (%)		4 (80)	1 (3.4)	0.001	112.0 (5.8, 2168.3)				

**Table 3:** Patient history and primary vital signs in mortality and intubation groups.

**Variable**		**Mortality**		***P*-value**	**Intubation**		***P*-value**
		**Yes (*n* = 5)**	**No (*n* = 29)**		**Yes (*n* = 5)**	**No (*n* = 29)**	
Age	Median	18	22	0.689^*^	37	21	0.234^*^
	IQR	13, 43.5	17, 28.5		14, 59	16.5, 28	
	Mean	26.2	23.6		36.8	21.8	
	SD	16.7	12.8		23.9	9.4	
	Min, max	10, 50	2, 69		10, 69	2, 41	
Admission days	Median	2	2	0.851^†^	2	2	0.851^†^
	IQR	1, 4.5	1, 3		1, 4.5	1, 3	
	Mean	2.6	2.3		2.6	2.3	
	SD	2.1	1.7		2.1	1.7	
	Min, max	1, 6.5	1, 8		1, 6	1, 8	
ICU duration day	Median	0	0	0.571^†^	0	0	0.571^†^
	IQR	0, 4	0, 1		0, 4	0, 1	
	Mean	1.6	0.6		1.6	0.6	
	SD	2.6	1.0		2.6	1.0	
	Min, max	0, 6	0, 4		0, 6	0, 4	
Colchicine dose of ingestion (mg)	Median	29	20	0.505	38	20	0.157
	IQR	17, 39.5	13, 30		18, 70	12, 28.7	
	Mean	28.5	26.8		42.8	24.2	
	SD	12.3	23.6		33.7	19.3	
	Min, max	16, 40	3, 100		16, 100	3, 100	
SBP mmHg	Median	90	110	0.011^†^	100	110	0.188^†^
	IQR	87.5, 100	100, 120		87.5, 110	100, 120	
	Mean	93	109		99	107.8	
	SD	6.7	11.8		13.4	12.2	
	Min, max	85, 100	85, 120		85, 120	85, 120	
DBP mmHg	Median	65	70	0.037^†^	65	70	0.3^†^
	IQR	62.5, 67.5	67.5, 80		62.5, 75	65, 80	
	Mean	65	72.2		68	71.6	
	SD	3.5	7.2		7.6	7.2	
	Min, max	60, 70	60, 80		60, 80	60, 80	
PR	Median	99	96	0.93^*^	88	96	0.672^*^
	IQR	80.5, 110	86, 110		82, 110	86.5, 111	
	Mean	96.5	97.2		94.4	97.6	
	SD	16.1	15.6		14.7	15.7	
	Min, max	78, 110	70, 130		78, 110	70, 130	
RR	Median	17	16	0.69^†^	16	16	0.609^†^
	IQR	13, 18	15, 20.5		14, 18	15, 20.7	
	Mean	16	17.8		16	17.9	
	SD	2.8	4.6		2.4	4.6	
	Min, max	12, 18	11, 31		12, 18	11, 31	
T centigrade	Median	37.2	37	0.472^†^	37.2	37	0.643^†^
	IQR	37.05, 37.3	37, 37.3		37, 37.3	37, 37.4	
	Mean	37.2	37.1		37.2	37.1	
	SD	0.1	0.3		0.1	0.3	
	Min, max	37, 37.3	36.5, 38		37, 37.3	36.5, 38	
O_2_ Sat percentage	Median	95.50	97	0.119^†^	96	97	0.338^†^
	IQR	95, 96.75	96, 98		95, 97.5	97, 0	
	Mean	95.75	96.76		96.2	96.7	
	SD	0.957	1.921		1.3	1.9	
	Min, max	95, 97	91, 100		95, 98	91, 100	

* *Applying t-test*;

† *Applying Mann-Whitney U-test*.

Significant correlation was present between abdominal pain and limb numbness with both mechanical ventilation and mortality. All patients who did not survive as well as 80% of the mechanical ventilation patients reported abdominal pain on arrival (*p* = 0.003, OR [95% CI] 2.2 [4.1, 7.9], and *p* = 0.04, OR [95% CI] 10.5 [1.1, 108.7], respectively). In the mortality and mechanical ventilation group, 60% of patients reported limb numbness, drowsiness, or tingling (*p* = 0.05, OR [95% CI] 18.7 [1.3, 272.1]). Dyspnea at presentation had significant association with mechanical ventilation. Almost 60% of intubated patients had shortness of breath on presentation (*p* = 0.006, OR [95% CI] 42.0 [2.9, 612.3]). 80% of those who died required mechanical ventilation. Low systolic and diastolic blood pressure (<90 and 60 mmHg, respectively) were significantly related to mortality (*p* = 0.029, OR [95% CI] 13.0 [1.5, 111.8]). 60% of patients who needed mechanical ventilation had a positive family history of rheumatoid arthritis (*p* = 0.029, OR: 13.0 [1.5, 111.8]). Dose of ingested colchicine has no significant correlation with mortality in this study.

### Laboratory Findings

Patients with poor outcomes had higher blood urea nitrogen (BUN) and creatinine, creatine phosphokinase (CPK) and lactate dehydrogenase (LDH), liver function tests, and international normalized ratio (INR) (Table 4).

**Table 4:** Lab test results in patients who eventually underwent intubation and those who were expired.

**Variable**		**Mortality**		***P*-value**	**Intubation**		***P*-value**
		**Yes (*n* = 5)**	**No (*n* = 29)**		**Yes (*n* = 5)**	**No (*n* = 29)**	
Sodium (mEq/L)	Median	144	140	0.185^*^	143	140	0.464^*^
	IQR	137, 146.5	138, 142		136, 146	138, 142	
	Mean	142.5	140.0		141.4	140.1	
	SD	5.3	3.1		5.2	3.1	
	Min, max	135, 147	131, 146		135, 147	131, 146	
Potassium (mEq/L)	Median	3.4	3.85	0.007^†^	3.5	3.9	0.003^†^
	IQR	3.1, 3.6	3.6, 4.5		3.15, 3.55	3.7, 4.5	
	Mean	3.3	4.1		3.4	4.1	
	SD	0.3	0.6		0.2	0.6	
	Min, max	3, 3.6	3.1, 5.7		3, 3.6	3.1, 5.7	
BUN (mg/dL)	Median	39	30	0.008^†^	38	28	0.013^†^
	IQR	35.5, 42.2	19, 32.5		33, 41.5	19, 32.7	
	Mean	39	27.7		37.4	27.6	
	SD	3.4	14.2		4.6	14.5	
	Min, max	35, 43	0.90, 76		31, 43	0.9, 76	
Creatinine (mg/dL)	Median	1.4	0.9	0.008^†^	1.4	0.9	0.034^†^
	IQR	1.1, 1.55	0.8, 1.1		0.9, 1.5	0.8, 1.1	
	Mean	1.35	0.9466		1.3	0.9	
	SD	0.25166	0.22198		0.3	0.2	
	Min, max	1, 1.6	0.5, 1.5		0.9, 1.6	0.5, 1.5	
CPK (U/L)	Median	1,364	141	0.087^†^	1,051	140.5	0.042^†^
	IQR	154, -	108, 6		300, 3335	102, 417.5	
	Mean	1836.7	443.3		1562	426.9	
	SD	1962.2	691.1		1693.669	707.3	
	Min, max	154, 3,992	53, 2,800		154, 3,992	53, 2,800	
CKMB (U/L)	Median	-	19	-		19	-
	IQR	-	8, -			8, -	
	Mean	-	688.3			688.3	
	SD	-	1168.8			1168.8	
	Min, max	-	8, 2,038			8, 2,038	
Troponin (ng/mL)	Median	0.9	0.29	-	0.9	0.29	
	IQR	0.9, 0.9	0.08, -		0.9, 0.9	0.08, -	
	Mean	0.9	0.29		0.9	0.29	
	SD	-	0.30		-	0.30	
	Min, max	0.9, 0.9	0.08, 0.5		0.9, 0.9	0.08, 0.5	
LDH (U/L)	Median	3169.5	523.5	0.029^†^	1,608	505	0.044^†^
	IQR	1228.5, 6012.7	406.5, 1290.5		1,006, 5585.5	406, 1,317	
	Mean	3470.2	1197.1		2958.2	1212.3	
	SD	2548.6	1541.1		2486.4	1581.8	
	Min, max	1,102, 6,440	283, 5,840		910, 6,440	283, 5,840	
Phosphorus (mg/dL)	Median	2.8	5.3	0.42^*^	2.8	5.3	0.42^*^
	IQR	2.8, 2.8	3.2, 8.1		2.8, 2.8	3.2, 8.1	
	Mean	2.8	5.6		2.8	5.6	
	SD	-	2.6		-	2.6	
	Min, max	2.8, 2.8	2.6, 9		2.8, 2.8	2.6, 9	
Calcium (mg/dL)	Median	7	8.8	0.045^*^	7	8.8	0.084^*^
	IQR	6.5, -	8, 9.7		6.5, -	8, 9.7	
	Mean	7	8.84		7	8.84	
	SD	0.70711	0.85323		0.70711	0.85323	
	Min, max	6.5, 7.5	8, 9.7		6.5, 7.5	8, 9.8	
Magnesium (mg/dL)	Median	1.9	1.9	-	1.9	1.9	-
	IQR	1.9, 1.9	1.8, -		1.9, 1.9	1.8, -	
	Mean	1.9	1.9		1.9	1.9	
	SD	-	0.14142		-	0.14142	
	Min, max	1.9, 1.9	1.8, 2		1.9, 1.9	1.8, 2	
Blood sugar (mg/dL)	Median	131.5	87.5	0.741^*^	106	87	0.632^*^
	IQR	52, -	78.7, 108.2		52, -	77, 109	
	Mean	131.5	97.3		123	96.7	
	SD	112.4	25.6		80.8	26.4	
	Min, max	52, 211	68, 161		52, 211	68, 161	
WBC (10^9^ cells/L)	Median	10,150	7,800	1^†^	10,150	7,800	1^†^
	IQR	300, -	6,000, 11,900		300, -	6,000, 11,900	
	Mean	10,150	9478.9		10,150	9478.9	
	SD	13930.00359	4748.51777		13930.0	4748.5	
	Min, max	300, 20,000	4,800, 20,200		300, 20,000	4,800, 20,200	
Platelet (cells/L)	Median	137,500	232,000	0.243^*^	137,500	232,000	0.243^*^
	IQR	12,000, -	172,000, 287,000		12,000, -	172,000, 287,000	
	Mean	137,500	225031.5789		137,500	225031.6	
	SD	177483.80208	91167.10574		177483.8	225031.6	
	Min, max	12,000, 263,000	13,600, 350,000		12,000, 263,000	13,600, 350,000	
Hemoglobin (mg/dL)	Median	9.9	13.6	0.286^†^	9.95	13.6	0.286^†^
	IQR	6.5, -	12.6, 16.0		6.5, -	12.6, 16	
	Mean	9.9	14.1		9.9	14.1	
	SD	4.9	2.2		4.9	2.2	
	Min, max	6.5, 13.4	12.6, 16		6.5, 13.4	10.2, 20.2	
AST (IU/L)	Median	507	46.5	0.008^†^	507	46.5	0.008^†^
	IQR	91, -	31.5, 76.25		91, -	31.5, 76.2	
	Mean	379.3	78.1		379.3	78.1	
	SD	250.2	88.2		250.2	88.2	
	Min, max	91, 540	14, 343		91, 540	14, 343	
ALT (IU/L)	Median	67	29	0.143^*^	67	29	0.143^*^
	IQR	60, -	19, 55		60, -	19, 55	
	Mean	67	38.8		67	38.8	
	SD	9.9	25.4		9.9	25.4	
	Min, max	60, 74	12, 100		60, 74	12, 100	
Alkaline phosphatase	Median	1530	229	0.002^†^	1,530	229	0.002^†^
	IQR	721, -	187.5, 392		721, -	187.5, 392	
	Mean	1461	295.5882		1461	295.6	
	SD	708.02613	173.71135		708.0	173.7	
	Min, max	721, 2,132	100, 665		721, 2,132	100, 665	
PT	Median	-	13.2	-	12	13.3	0.462^†^
	IQR	-	12.1, 14.3		12, 12	12.4, 14.4	
	Mean	-	13.9		12	14.1	
	SD	-	2.4		-	2.5	
	Min, max	-	11.8, 19.2		12, 12	11.8, 19.2	
PTT	Median	45	31	0.001^*^	42	31	0.021^*^
	IQR	39, -	27.6, 35		30.7, 50.2	27.6, 35.2	
	Mean	45.3	31.4		41	31.7	
	SD	6.5	4.8		10.2	4.9	
	Min, max	39, 52	25, 41		28, 52	25, 41	
INR	Median	4	1.1	0.023^†^	3.5	1.2	0.185^†^
	IQR	3.1, -	1, 1.9		1.5, 4.0	1, 2.0	
	Mean	3.7	2.7		3.0	2.8	
	SD	0.6	5.4		1.4	5.6	
	Min, max	3.07, 4.06	0.9, 23		1, 4.1	0.9, 23	
pH (venous)	Median	7.34	7.36	0.395^†^	7.28	7.36	0.096^†^
	IQR	7.23, -	7.34, 7.41		7.1175, 7.37	7.34, 7.41	
	Mean	7.32	7.36		7.26	7.38	
	SD	0.08	0.09		0.13	0.06	
	Min, max	7.23, 7.38	7.08, 7.51		7.08, 7.38	7.26, 7.51	
pCO2 (venous)	Median	31	40	0.594^†^	40.5	40	0.706^†^
	IQR	12, -	30.1, 45		16.7, 104.7	30.1, 45	
	Mean	31	41.7522		54	38.1	
	SD	19	19.54377		48.5	8.4	
	Min, max	12, 50	18, 123		12, 123	18, 50	
HCO3 (venous)	Median	20	24	0.04^*^	20.5	23.9	0.818^*^
	IQR	7.1, -	21.2, 25		10.3, 32.1	20.9, 25	
	Mean	16.0	23.0		21.0	22.5	
	SD	7.7	4.9		11.7	4.2	
	Min, max	7.1, 21	12.4, 35.8		7.1, 35.8	12.4, 29	

* *Applying t-test*;

† *Applying Mann-Whitney U-test*.

The odd [95% CI] of 84 times [4.1, 1715.6] for death was achieved using the cut-off of 1.35 mg/dl for creatinine level (*p* = 0.003). It was 17 [1.3, 223.1] for LDH ≥ 1,551 (*p* = 0.035), 5 [2.1, 12.0] for AST ≥ 85 mg/dl (*p* = 0.02), 8 [2.2, 29.2] for INR ≥ 2.64 (*p* = 0.01), 7.2 [2.9, 18.0] for BUN ≥ 34.5 (*p* = 0.002), and 7 [1.9, 25.2] for PTT ≥ 37.5 s (*p* = 0.015).

Bicarbonate level (but not pH) was significantly lower in the mortality group and so were serum potassium and calcium levels (Table 4).

### Electrocardiographic Findings

Point of care ECGs for 27 patients were available. Thirteen (38%) were male and median [IQR] age was 22 years. Five patients were intubated and five passed away during the hospital stay. All patients had some ECG abnormalities. Sinus tachycardia and bradycardia were detected in 10 (37%) and one patient, respectively. Mean heart rate was significantly higher in patients who were eventually intubated or died (*p* = 0.02). One patient was reported to have premature ventricular complexes (PVC). Other abnormalities seen were left atrial enlargement in 13 patients (48%), PR elevation in aVR lead in 10 (37%), pathologic Q wave in 10 (37%), ST segment elevation in 7 (37%), ST segment depression in 9 (33%), Brugada pattern in 6 (22%), T wave inversion in 10 (37%), and pathologic U wave in 2 (7%). None of these changes were related to mechanical ventilation and death in a statistically significant manner. ECG abnormalities reported are summarized in Tables 5, 6.

**Table 5:** Qualitative ECG findings in mortality and intubation groups.

**Variable**		**Mortality**		***P*-value**	**Intubation**		***P*-value**
		**Yes (*n* = 5)**	**No (*n* = 22)**		**Yes (*n* = 5)**	**No (*n* = 22)**	
Rhythm	Normal sinus *n* (%)	1(25)	15 (65.2)	0.231	1 (25)	15 (65)	0.231
	Sinus Tachycardia *n* (%)	3(75)	7 (30.4)		3 (75)	7 (30)
	Sinus Bradycardia *n* (%)	0	1 (4.3)		0	1 (4.3)
PVC		0	1 (4.3)	1	0	1 (4.3)	1
PAC							
AXIS	NAD *n* (%)	2 (50)	20 (87)	0.187	2 (50)	20 (87)
	RAD *n* (%)		1 (25)	2 (8.7)	1 (25)	2 (8.7)	0.187
	LAD *n* (%)		1 (25)	1 (4.3)	1 (25)	1 (4.3)
LAHB *n* (%)							
LPHB *n* (%)		1 (25)	2 (8.7)	0.395	1 (25)	2 (8.7)	0.395
LAA *n* (%)		1 (25)	12 (52)	0.596	1 (25)	12 (52)	0.596
RAA *n* (%)							
WPW *n* (%)							
PR_Elevation_Lead_aVR *n* (%)		2 (50)	8 (34)	0.613	2 (50)	8 (34)	0.613
PR Depression *n* (%)		1 (25)	9 (39)	1	1 (25)	9 (39)	1
PR Depression Lead I *n* (%)		0	2 (8.7)	1	0	2 (8.7)
PR Depression Lead II *n* (%)		1(25)	5 (21)	1	1 (25)	5 (21)	1
PR Depression Lead III *n* (%)		0	1 (4.3)	1	0	1 (4.3)	1
PR Depression Lead V3 *n* (%)		1 (25)	1 (4.3)	0.279	1 (25)	1 (4.3)	1
PR Depression Lead V4 *n* (%)		1 (25)	2 (8.7)	0.395	1 (25)	2 (8.7)	0.279
PR Depression Lead V5 *n* (%)		1 (25)	3 (13)	0.495	1 (25)	4 (14.8)	0.395
PR Depression Lead V6 *n* (%)		1 (25)	2 (8.7)	0.395	1 (25)	2 (8.7)	0.495
PR Depression Lead avr *n* (%)		1 (25)	6 (26)	1	1 (25)	6 (26)	0.395
AV Block		0	1 (4.3)	1	0	1 (4.3)	1
LBBB		0	2 (8.7)	1	0	2 (8.7)	1
RBBB							
Pathologic Q Wave		0	10 (43)	0.264	0	10 (43.5)	0.264
Pathologic Q Wave Lead II		0	6 (26)	0.545	0	6 (26)	0.545
Pathologic Q Wave Lead III		0	8 (34.8)	0.285	0	8 (34.8)	0.285
Pathologic Q Wave Lead avf		0	4(17.4)	1	0	4 (17.4)	1
Pathologic Q Wave Lead avr		0	2 (8.7)	1	0	2 (8.7)	1
Pathologic Q Wave avl		0	2 (8.7)	1	0	2 (8.7)	1
Pathologic Q Wave_Lead V5		0	1 (4.3)	1	0	1 (4.3)	1
Pathologic Q Wave Lead V6		0	1 (4.3)	1	0	1 (4.3)	1
Tall R		1 (25) 2 (8.7)	0.395	1 (25) 2 (8.7)	0.395
Tall R_LEAD V1		1 (25) 0	0.148	1 (25) 0	0.148
Tall R_LEAD V2		1 (25) 0	0.148	1 (25) 0	0.148
Tall R_LEAD V4		0	1 (4.3)	1	0	1 (4.3)	1
Tall R_LEAD V5		0	2 (8.7)	1	0	2 (8.7)	1
Tall R_LEAD V6		0	2 (8.7)	1	0	2 (8.7)	1
Deep S		1 (25) 1 (4.3)	0.279	1 (25) 1 (4.3)	0.279
Deep S LEAD V3		0	1 (4.3)	1	0	1 (4.3)	1
Deep S LEAD V5		1(25)	0	0.148	0	1(3.7)	0.148
Deep S LEAD V6		1(25)	0	0.148	1 (25) 0	0.148
LVH		0	1 (4.3)	1	0	1 (4.3)	1
RVH							
Poor R Progression		1 (25)	5(21.7)	1	1(25)	5 (21)	1
Poor R Progression Lead_V1		0	4 (17.4)	1	0	4 (17.4)	1
Poor R Progression Lead V2		0	4 (17.4)	1	0	4 (17.4)	1
Poor R Progression Lead V3		0	4 (17.4)	1	0	4 (17.4)	1
Poor R Progression Lead V4		0	1 (4.3)	1	0	1 (4.3)	1
ST Elevation		1 (25) 6 (26.1)	1	1 (25)	6 (26)	1
ST Elevation Specific		1 (25) 5 (21.7)	1	1 (25)	5 (21)	1
ST Elevation Type		4 (100) 19 (82.6) 1	4 (100)	19 (82)	1
ST Elevation Lead V1		0	2 (8.7)	1	0	2 (8.7)	1
ST Elevation Lead V2		0	1 (4.3)	1	0	1 (4.3)	1
ST Elevation Lead V3		0	2 (8.7)	1	0	2 (8.7)	1
ST Elevation Lead V4		0	2 (8.7)	1	0	2 (8.7)	1
ST Elevation Lead V5		0	1 (4.3)	1	0	1 (4.3)	1
ST Elevation Lead V6		0	1 (4.3)	1	0	1 (4.3)	1
ST Elevation Lead I		0	1 (4.3)	1	0	1 (4.3)	1
ST Elevation Lead avr		1 (25)	3 (13)	0.495	1 (25) 3 (13)	1
ST Elevation Lead avl		0	1 (4.3)	1	0	1 (4.3)	0.495
ST Depression		1 (25)	8 (34.8)	1	1 (25)	8 (34.8)	1
ST Depression Type		4 (100)	15 (65.2) 0.285	4 (100)	15 (65.2)	1
ST Depression Lead V1		0	1 (4.3)	1	0	1 (4.3)	0.285
ST Depression Lead V4		0	1 (4.3)	1	0	1 (4.3)	1
ST Depression Lead V5		1 (25)	3 (13)	0.495	1 (25) 3 (13)	1
ST Depression Lead V6		1 (25)	3 (13)	0.495	1 (25)	3 (13)	0.495
ST Depression Lead I		0	1 (4.3)	1	0	1 (4.3)	0.495
ST Depression Lead II		0	4 (17.4)	1	0	4 (17.4)	1
ST Depression Lead III		0	4 (17.4)	1	0	4 (17.4)	1
ST Depression Lead avr		0	2 (8.7)	1	0	2 (8.7)	1
ST Depression Lead avf		0	1 (4.3)	1	0	1 (4.3)	1
ST Changes		1 (25)	11 (47.8) 0	0.605	1 (25)	11 (47.8)	1
J Point Elevation		0	3 (13)	1	0	3 (13)	0.605
J_Point_Elevation_Lead_V2		0	1 (4.3)	1	0	1 (4.3)	1
J_Point_Elevation_Lead V3		0	1 (4.3)	1	0	1 (4.3)	1
J_Point_Elevation_Lead V4		0	1 (4.3)	1	0	1 (4.3)	1
J_Point_Elevation_Lead V5		0	1(4.3)	1	0	1(4.3)	1
J_Point_Elevation_Lead I		0	1 (4.3)	1	0	1 (4.3)	1
J_Point_Elevation_Lead II		0	1 (4.3)	1	0	1 (4.3)	1
Early Repolarization Lead V2_V3_V4_V5 0		1 (4.3)	1	0	1 (4.3)	1
Brugada like syndrome rSr TYPE		0	6 (26)	0.545	0	6 (26)	1
Brugada_like_syndrome_Lead_V1		0	5 (21.7)	0.561	0	5 (21.7)	0.545
Brugada like syndrome Lead V2		0	1 (4.3)	1	0	1 (4.3)	0.561
T Wave inversion		0	10 (43.5) 0.264	0	10 (43.5)	1
T Wave inversion Lead V1		0	4 (17.4)	1	0	4 (17.4)	0.264
T Wave inversion Lead V2		0	4 (17.4)	1	0	4 (17.4)	1
T Wave inversion Lead V3		0	5 (21.7)	0.561	0	5 (21.7)	1
T Wave inversion Lead V4		0	3 (13)	1	0	3 (13)	0.561
T_Wave_inversion_Lead_V5		0	1 (4.3)	1	0	1 (4.3)	1
T Wave inversion Lead I		0	1 (4.3)	1	0	1 (4.3)	1
T Wave inversion Lead III		0	4 (17.4)	1	0	4 (17.4)	1
T Wave inversion Lead avl		0	1 (4.3)	1	0	1 (4.3)	1
T Wave inversion Lead avr		0	1 (4.3)	1	0	1 (4.3)	1
T Wave Wide Lead V3_V4_V5_V6		0	1 (4.3)	1	0	1 (4.3)	1
U wave		0	4 (17.4)	1	0	4 (17.4)	1
U wave Pathologic		0	2 (8.7)	1	0	2 (8.7)	1
U wave LEAD V3		0	2 (8.7)	1	0	2 (8.7)	1
U wave LEAD V4		0	2 (8.7)	1	0	2 (8.7)	1
U wave LEAD V5		0	1 (4.3)	1	0	1 (4.3)	1
TDP
1 Dynamic Change		1 (25)	5 (21.7)	1	1 (25)	5 (21.7)	1
Dynamic Change Description	without ST changes, PR changes and T inversion in v3, v4	0	1 (4.3)		0	1 (4.3)	
	tall R, rSr' in v1, pathologic U in inf. leads, LAA in days after 0	1 (4.3)		0	1 (4.3)	
	RSR' changes to Rs in v1 without QRS widness	0	1 (4.3)		0	1 (4.3)	
	VT then Slow VT then Asystole in next days	1 (25)	0	0.357	1 (25)	0	0.357
	T wave + in 3rd day in v3-v4 and decreased AR to 70.0 also T inversion is age related in this case 0	1 (4.3)		0	1 (4.3)	
	ST Depression in v2, v3, v4, v5, v6, limb2, limb3, aVF in the 3rd day of admission 0	1 (4.3)		0	1 (4.3)	

**Table 6:** Quantitative ECG findings in mortality and intubation groups.

**Variable**		**Mortality**		***P*-value**	**Intubation**		***P*-value**
		**Yes (*n* = 5)**	**No (*n* = 22)**		**Yes (*n* = 5)**	**No (*n* = 22)**	
QTc (ms)	Median	426.5	402	0.201^*^	426.5	402	0.201^*^
	IQR	402.7, 448.7	390, 429		402.5, 448.5	390, 429	
	Mean	426	407.1087		426	407.1087	
	SD	23.8	26.9		23.8	26.9	
	Min, max	398, 453	355, 464		398, 453	355, 464	
ECG rate (/min)	Median	110	91	0.025^*^	110	91	0.025^*^
	IQR	96, 131	79, 105		96, 130.7	79, 105	
	Mean	112.2	89.6		112.2	89.6	
	SD	18.2	17.5		18.2	17.5	
	Min, max	93, 136	40, 115		93, 1	40, 1	
PR interval (ms)	Median	0.1	0.2	0.216^†^	0.1	0.2	0.216^†^
	IQR	0.09, 0.16	0.12, 0.16		0.1, 0.1	0.1, 0.2	
	Mean	0.12	0.15		0.12	0.14	
	SD	0.03	0.02		0.03	0.02	
	Min, max	0.08, 0.16	0.12, 0.2		0.08, 0.16	0.12, 0.2	
QRS duration (ms)	Median	0.08	0.08	0.448^†^	0.08	0.08	0.448^†^
	IQR	0.07, 0.08	0.08, 0.09		0.07, 0.08	0.08, 0.09	
	Mean	0.08	0.08		0.08	0.08	
	SD	0.01	0.01		0.01	0.01	
	Min, max	0.07, 0.08	0.05, 0.11		0.07, 0.08	0.05, 0.11	

* *Applying t-test*;

† *Applying Mann-Whitney U-test*.

Three of the cases (11%) had prolonged QTc interval. Mean QTc [IQR] was 426 ms [402.75 to 448.75 ms] in the mechanical ventilation and mortality groups (*p* = 0.20). QTc, PR interval, and QRS duration were not significantly different between mortality and survival groups.

There were 13 cases of left atrial enlargement and no cases of right atrial enlargement. PR elevation in lead aVR and PR depression in the limb leads, anterolateral leads, and aVR leads were found in 10 patients. Inverted T waves and pathological Q wave in II and III were seen in 10 cases each. ST-elevation was detected in 7 patients in precordial leads, I, aVR, and aVL. Brugada-like pattern was recorded in six cases in leads V1 and V2. Poor R progression was detected in 6 ECGs in the anteroseptal precordial leads. Three cases of left posterior hemiblock were detected. Pathological U waves and left bundle branch block were detected in two cases. J point elevation, first degree atrioventricular block, and early repolarization were present in one case each.

Dynamic ECG changes were noted in six patients during their hospital stay. Except for the heart rate, none of the ECG variables were found to be related to mortality and intubation. Mean heart rate was 112/min (range: 93-136/min) and 90/min (range: 40-115/min) in the mortality and mechanical ventilation groups, respectively. Mode of death was bradycardia/asystole in 4, and ventricular tachycardia in 1 patient.

## Discussion

Previous studies on colchicine poisoning are generally case reports and small case series (17–19). The clinical effects of colchicine on different organs are shown in Table 7. In this study, we evaluated clinical presentation, detailed ECG abnormalities, laboratory data on arrival, and outcome of 34 colchicine-intoxicated patients who were referred to our tertiary toxicology center during a 10-year period.

**Table 7:** General involvements of different organ systems in colchicine poisoning (phases of colchicine poisoning).

**Phase**	**Time**	**Signs and symptoms**
I	0–24 h	Gastrointestinal (nausea/vomiting/diarrhea) Leukocytosis
II	1–7 days	Risk of sudden cardiac death Pancytopenia Acute kidney injury' Sepsis Acute respiratory distress syndrome Electrolyte imbalance Rhabdomyolysis
III	>7 days	Alopecia Myopathy Neuropathy Myoneuropathy

Although most of the ECG abnormalities we found in our study were not significantly related to the patient's outcome, our detailed findings seem to be singular. Previously reported cases had shown ST elevation in patients with colchicine poisoning (20). Bradycardia and arrhythmia had been reported in severe toxicities after intravenous administration of colchicine (21). However, to the best of our knowledge, no studies have clearly detailed the ECG effects of colchicine in intoxicated patients. Colchicine effect on skeletal muscles has been discussed (22).

Clinical results are also of importance. Based on our findings, tachycardia is significantly related with intubation and mortality in colchicine-intoxicated patients; a finding that had never been reported before. Troponin level does not significantly change after colchicine poisoning although cardiac changes are common showing the fact that the nature of the damage to the cardiac cells is not ischemic or necrosis-inducing in this poisoning.

Also, previous studies have reported laboratory abnormalities in colchicine poisoning, but none had evaluated the relation of these abnormalities with mortality (23). High BUN and Cr and lower serum potassium and calcium significantly increased the risk of mortality. Renal failure, hypokalemia, and hypocalcemia are due to diarrhea and GI loss. Metabolic acidosis was another important laboratory finding associated with mortality in these patients.

If rhabdomyolysis occurs, hemoglobinuria may also cause azotemia (24). This can be considered a possible cause of azotemia in four of our patients who had CPK of more than 1,000 mg/dl and higher mortality. Arslan et al. reported a case of colchicine poisoning with evidence of myocytolysis in autopsy (25) confirming the possible effect of the drug on myocytes. Colchicine has been known to induce myopathy in several other studies, as well (26, 27).

Similar to previous studies, the most common presentation of our patients were GI manifestations including nausea, vomiting, abdominal pain and diarrhea (8, 10) which can occur due to mucosal damage and cholera-like syndrome (28). Elevated liver enzymes and higher INR in our cases were significantly related to mortality. We think hypovolemia and hypotension might have played a role through ischemic hepatitis. Dyspnea was an important presentation of our patients and was significantly related to intubation. Case reports of acute respiratory distress syndrome due to colchicine poisoning exist in the literature but none of our patients had ARDS (29). Direct toxic effect of colchicine on respiratory muscles have already been suggested as the mechanism of respiratory involvement (30, 31) which can be argued through our results, as well.

Neuropathy was not prominent in our patients. Limb and facial numbness and paresthesia, headache, tremor, and ataxia were the most common presentations. Patients who complained of numbness, tingling, and facial paresthesia on admission had a higher risk of mortality and need for intubation. Different forms of neuropathy including myo-neuropathy, distal sensory abnormalities, nerve conduction impairment, and axonal neuropathy have been reported in previous studies and changes in microtubular network has been suggested as the probable mechanism of myopathy in these patients (7, 32).

Considering the results of the current study, BUN, creatinine, potassium, and calcium disturbances on presentation accompany with a poorer prognosis. Admission of patients with these problems to the intensive care unit and obsessive correction of the electrolyte and kidney disturbances may yield better prognosis in these patients.

### Limitations and Recommendations

Our main limitation was inability to check serum colchicine level and to correlate it with symptoms. Another limitation was retrospective nature of the study that led to some missing data.

The low prevalence of colchicine poisoning makes it difficult to conduct a prospective study with attention to levels and cardiac poisoning. Therefore, we recommend more close attention to ECG changes, hemodynamics, and laboratory abnormalities in colchicine toxic patients to detect high risk patients who are at risk of intubation and death.

## Conclusion

Colchicine toxicity is a potentially dangerous situation that needs close monitoring and management. Abdominal pain, sinus tachycardia, hypotension, elevated renal function tests and low potassium are signs of danger and might require intensive care to avoid mechanical ventilation and mortality.

## Data Availability Statement

The raw data supporting the conclusions of this article will be made available by the authors, without undue reservation.

## Ethics Statement

The study was reviewed and approved by Institutional Review Board of Shahid Beheshti University of Medical Sciences (IR.SBMU.MSP.REC.1398.116). The need for written informed consent was waived due to the retrospective nature of the study.

## Author Contributions

MS developed the concept, made the questionnaire, wrote the manuscript, and made the tables. NZ developed the concept, edited the manuscript, and did critical thinking on the subject. AG collected the data and drafted the manuscript. HA edited the final manuscript. HH-M edited the manuscript, submitted it to the journal, and is responsible for the overall content as guarantor. All authors contributed to the article and approved the submitted version.

## Conflict of Interest

The authors declare that the research was conducted in the absence of any commercial or financial relationships that could be construed as a potential conflict of interest.

## Publisher's Note

All claims expressed in this article are solely those of the authors and do not necessarily represent those of their affiliated organizations, or those of the publisher, the editors and the reviewers. Any product that may be evaluated in this article, or claim that may be made by its manufacturer, is not guaranteed or endorsed by the publisher.
